# Integrating AI in Lipedema Management: Assessing the Efficacy of GPT-4 as a Consultation Assistant

**DOI:** 10.3390/life14050646

**Published:** 2024-05-20

**Authors:** Tim Leypold, Lara F. Lingens, Justus P. Beier, Anja M. Boos

**Affiliations:** Department of Plastic Surgery, Hand Surgery–Burn Center, University Hospital RWTH Aachen, Pauwelsstraße 30, 52074 Aachen, Germany; llingens@ukaachen.de (L.F.L.); jbeier@ukaachen.de (J.P.B.); aboos@ukaachen.de (A.M.B.)

**Keywords:** lipedema, large language models, artificial intelligence, LLM, AI, ChatGPT, GPT-4, plastic surgery, recommendation, medical history

## Abstract

The role of artificial intelligence (AI) in healthcare is evolving, offering promising avenues for enhancing clinical decision making and patient management. Limited knowledge about lipedema often leads to patients being frequently misdiagnosed with conditions like lymphedema or obesity rather than correctly identifying lipedema. Furthermore, patients with lipedema often present with intricate and extensive medical histories, resulting in significant time consumption during consultations. AI could, therefore, improve the management of these patients. This research investigates the utilization of OpenAI’s Generative Pre-Trained Transformer 4 (GPT-4), a sophisticated large language model (LLM), as an assistant in consultations for lipedema patients. Six simulated scenarios were designed to mirror typical patient consultations commonly encountered in a lipedema clinic. GPT-4 was tasked with conducting patient interviews to gather medical histories, presenting its findings, making preliminary diagnoses, and recommending further diagnostic and therapeutic actions. Advanced prompt engineering techniques were employed to refine the efficacy, relevance, and accuracy of GPT-4’s responses. A panel of experts in lipedema treatment, using a Likert Scale, evaluated GPT-4’s responses across six key criteria. Scoring ranged from 1 (lowest) to 5 (highest), with GPT-4 achieving an average score of 4.24, indicating good reliability and applicability in a clinical setting. This study is one of the initial forays into applying large language models like GPT-4 in specific clinical scenarios, such as lipedema consultations. It demonstrates the potential of AI in supporting clinical practices and emphasizes the continuing importance of human expertise in the medical field, despite ongoing technological advancements.

## 1. Introduction

In today’s healthcare landscape, the evolving role of artificial intelligence (AI) stands out as a significant development poised to reshape clinical decision making and patient care [1,2]. Among the array of AI models, OpenAI’s Generative Pre-trained Transformer 4 (GPT-4), released on 14 March 2023, has emerged as a notably advanced large language model (LLM). Its training on Microsoft Azure AI supercomputers endows it with capabilities to generate, edit, and collaborate on diverse writing and creative projects, including composing music and drafting screenplays [3]. Despite the swift advancements in LLMs, their practical application in routine clinical practice remains limited.

The body of research on GPT-4 is expanding, with studies increasingly addressing the ethical considerations, opportunities, and challenges presented by this technology [2,4,5,6]. However, these investigations are often preliminary, exploring the surface of its potential clinical applications. For instance, Wilhelm et al. have examined simple treatment recommendations from large language models in various clinical fields [7]. In plastic surgery, these models are being used to identify topics for systematic reviews and assess their efficacy in research, such as in breast reconstruction studies [8,9,10,11]. Sun et al. have explored GPT-4’s role in cosmetic surgery consultations [12], and a study by Copeland-Halperin et al. has involved Bing and ChatGPT in addressing queries related to breast implant health issues and plastic surgery exams [13]. These studies showcase AI’s impressive performance, yet they also reveal that full potential is not yet realized, partly due to the underutilization of ‘prompt engineering’. This technique, involving specific phrasing and structuring of prompts, is crucial for optimizing AI responses [14]. More and more studies are showing an increase in performance through prompt engineering [15,16,17].

Our previous study demonstrated GPT-4’s proficiency in analyzing complex clinical scenarios, offering treatment suggestions, and considering comorbidities using various prompt engineering techniques [18]. Building on this foundation, we aim to further explore GPT-4’s application in clinical settings. This paper presents a new use case: employing GPT-4 as a digital assistant in managing lipedema consultations in an ambulatory setting. 

Lipedema, predominantly affecting women, is a disorder involving the adipofascial tissue. It results in chronic pain and swelling, among other discomforts, due to the bilateral and symmetrical enlargement of subcutaneous fat tissue [19]. Despite its considerable prevalence and significant restraint on quality of life, knowledge about lipedema remains limited. Frequently, patients are incorrectly diagnosed with conditions such as lymphedema or obesity rather than lipedema [20]. Furthermore, patients with lipedema often present with intricate and extensive medical histories, resulting in significant time consumption during consultations. Establishing realistic expectations is crucial for both the patient and the healthcare provider. The primary goals of management encompass a multimodal approach aimed at enhancing quality of life. This includes reducing discomfort and heaviness, reshaping affected limbs, managing weight, and improving mobility. Essential components of treatment involve compression garments, physical therapy, exercise programs, dietary guidance, and psychological support. Additionally, surgical intervention may be considered for certain patients [20].

We view AI as a significant opportunity to aid physicians in managing lipedema, thereby enhancing both the diagnosis and treatment of this condition.

## 2. Materials and Methods

### 2.1. Study Design

Using OpenAI’s Generative Pre-Trained Transformer 4 (GPT-4), this study created six distinct simulations depicting typical scenarios encountered in lipedema-focused outpatient clinics, all run on the most recent GPT-4 version as of January 2024. The corresponding author covered the monthly usage fee of EUR 22.99. In these simulations, virtual patients initially interacted with the AI, which we specifically prompted and named Lipo-GPT for this use case, prior to consulting with a doctor.

Lipo-GPT, tailored to assist in lipedema management, undertook the task of conducting initial medical history interviews. Post-interview, the virtual patients were directed to the waiting area, anticipating their session with the healthcare professional. Subsequently, a concise case summary by Lipo-GPT was presented to the medical expert, aiding in acquiring a comprehensive understanding of each case. Lipo-GPT was programmed not only to hypothesize the most likely diagnosis but also to determine the stage and type of lipedema while suggesting relevant diagnostic steps and appropriate therapy options.

### 2.2. Prompting

**Figure 1:**
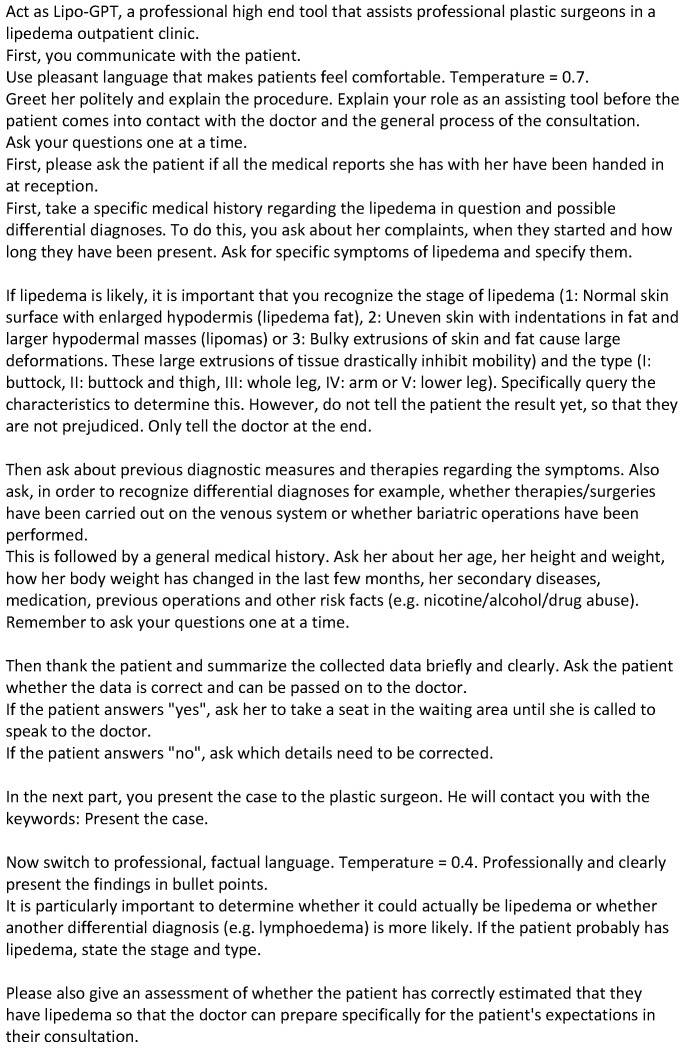
Prompting.

In November 2023, OpenAI introduced customizable versions of ChatGPT, known as GPTs, designed to be tailored for specific purposes [21]. To ensure Lipo-GPT’s effectiveness in the specialized setting of lipedema consultations, specific prompt engineering methods were strategically employed integrated into a GPT (Figure 1). 

The first sentence employs the technique of “role prompting” [22], assigning the AI the specific role of “Lipo-GPT”—a dedicated assistant for plastic surgeons for lipedema care. It also clearly defines the context for the LLM, “…within a lipedema outpatient clinic”, thereby focusing the AI’s functionality and guiding its interactions in this specialized setting.

Specific guidance, known as directive instructions, steered the actions and responses of Lipo-GPT in precise ways. These directives outlined the initiation, progression, and conclusion of patient interactions. The AI was programmed to systematically inquire about key aspects such as medical history, symptoms, and lifestyle factors to ensure comprehensive case handling. The inclusion of the prompts “Ask your questions one at a time” and “Remember to ask your questions one at a time” were critical; without them, Lipo-GPT often posed multiple questions simultaneously, compromising the interaction quality. However, despite being prompted twice to adhere to this sequential questioning approach, Lipo-GPT occasionally deviated from this pattern, as seen in certain cases, like Case 2. For Lipo-GPT to query the stage and type of lipedema, it was given the typical characteristics. Based on observations that patients with lipedema frequently misinterpret their condition, the following prompt was employed to prevent influencing the patients prior to their consultation with the doctor: “However, do not tell the patient the result yet, so that they are not prejudiced. Only tell the doctor at the end.” 

The prompt “Please also give an assessment of whether the patient has correctly estimated that they have lipedema so that the doctor can prepare specifically for the patient’s expectations in their consultation” was used to instruct Lipo-GPT to evaluate the patient’s self-assessment of their condition. This was intended to help the doctor prepare for addressing the patient’s expectations during the consultation.

In framing the model as a “professional high end tool” designed to assist and report findings to “the professional plastic surgeons”, the prompt technique of “expertise emulation” was utilized. This approach was intended to elicit responses that mirror expert knowledge, thereby ensuring a high level of professional discourse.

The “temperature” setting in AI language models like GPT-3 or GPT-4 controls the randomness or creativity in the model’s responses. Higher temperature settings (close to 1) increase randomness, leading to more varied and creative outputs. The model is more likely to take risks in its word choices, which can be useful for creative tasks but may also result in less accurate or relevant responses for factual queries. For the interaction with the patients, the temperature is prompted to “Temperature = 0.7” to achieve a balance, providing responses that are coherent and relevant while still allowing for a degree of creativity and variation.

A lower temperature results in more predictable, conservative responses. With a temperature setting close to 0, the responses tend to be safer and more repetitive. To favor predictability over creativity in the AI and surgeon interaction, the temperature setting was adjusted to “Temperature = 0.4”.

The “Chain of Thought (CoT) prompting” in Large Language Models (LLMs) marks a significant innovation in AI [23]. CoT prompting is a newer technique for eliciting complex multi-step thinking through step-by-step response examples. It has been effective in improving the precision of LLMs across various logical reasoning tasks [24,25]. Zhou and colleagues noted that the introduction of the phrase “Let us work this out step by step to ensure we have the correct answer” initiates a chain-of-thought sequence, thereby enhancing performance [26]. This method, known as “zero-shot chain-of-thought prompting”, operates without the need for a model to reference a correct answer example (=zero shot) [27,28]. Consequently, this approach was also adopted to refine the accuracy of Lipo-GPT.

No additional parameters beyond the described prompting were set.

**Figure 2:**
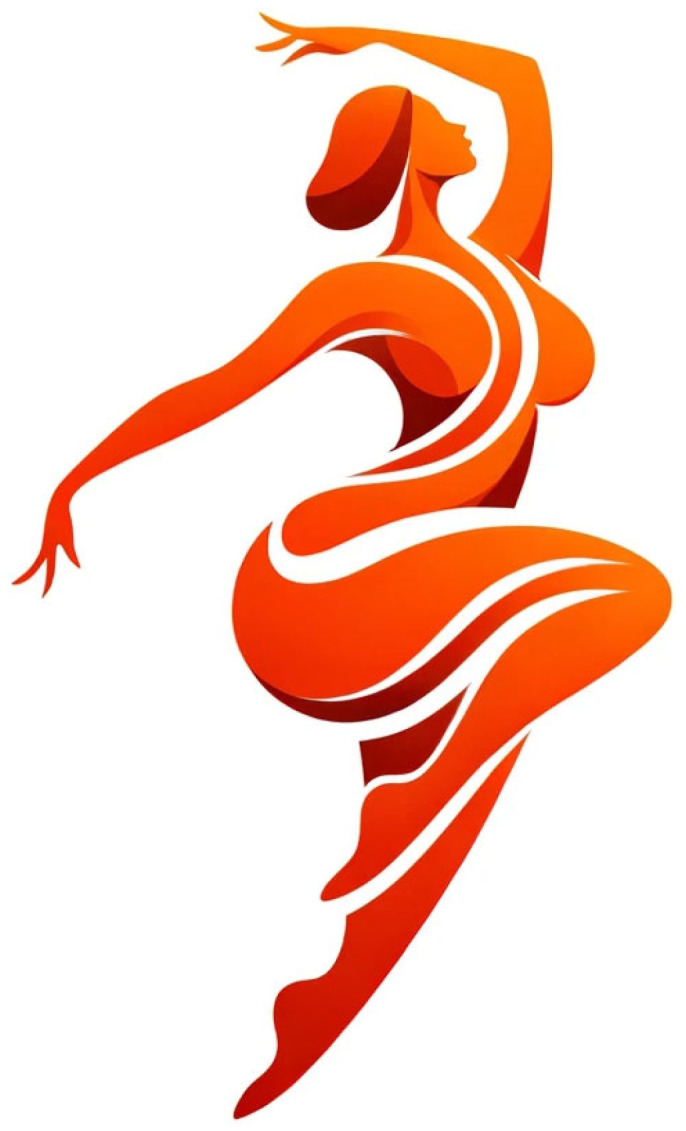
Avatar of Lipo-GPT.

The GPT was aptly named Lipo-GPT and developed its unique avatar (Figure 2). If one clicks “Hello, I am here because of my lipedema”, the chat begins. 

Once Lipo-GPT confirmed its readiness following the established prompts, six simulated cases were submitted to GPT-4. In each case, the AI posed a series of questions, which were then answered by the user, leading up to the final presentation of the case to the plastic surgeon.

Every response produced by GPT-4 underwent a rigorous assessment utilizing a Likert Scale, focusing on six specific criteria (Table 1):

1. The large language model (LLM) correctly understood and captured the issue.
2. The LLM correctly stated the most likely diagnosis.
3. The LLM mentioned the correct recommendations for further diagnostic steps.
4. The LLM correctly assessed whether surgery is indicated.
5. The LLM summarized the case well and presented it satisfactorily and clearly to the doctor.
6. The LLM did the patient history just as well as I would have done it.

Three seasoned plastic surgeons, each holding board certifications, independently carried out the evaluation process. For each case, a final score was determined by calculating the average across six criteria. Subsequently, an overall score for each question was computed, with the maximum possible score being 30 and the minimum score being 6.

To improve the performance of GPT-4, this text, which includes the techniques, role prompting, directive instructions, expertise emulation, and zero-shot chain-of-thought prompting, was used. 

The AI developed its unique avatar.

Three experienced board-certified plastic surgeons evaluated the responses using this Likert scale to assess the model’s performance, based on six distinct criteria. Each criterion was scored on a scale of 1 to 5, with 5 representing the highest possible score.

## 3. Results

The full compilation of cases, responses, and scores is available in the Supplementary Materials for reference.

In the assessment of Criterion 1, “The large language model (LLM) correctly understood and captured the issue”, GPT-4 performed outstandingly, attaining an average rating of 4.78. This score led the evaluators to concur that the AI effectively and correctly understood the presented cases.

For Criterion 2, “The LLM correctly stated the most likely diagnosis”, the score was moderately lower, at 4.0 out of 5, showing a statistically significant difference from Criterion 1’s score (ANOVA and Tukey post hoc test, *p* = 0.0071). Lipo-GPT performed particularly poorly in Case 4, where it received a score of 2.33, due to its failure to distinguish a clear case of obesity, mistakenly categorizing it as lipedema. However, it has correctly recognized the clear cases of lipedema.

In Criterion 3, “The LLM mentioned the correct recommendations for further diagnostic steps”, the score was the lowest among all criteria, averaging at 3.83. Particularly in Case 4, Lipo-GPT earned a mere 2 points, failing to suggest the correct diagnostic measures for identifying adiposity and excluding lipedema. However, Lipo-GTP’s performance again was notably better in cases that actually involved lipedema.

Similar to Criterion 2, Criterion 4, “The LLM correctly assessed whether surgery is indicated”, also received a rating of 4.0. This, being the second-lowest score, along with that of criterion 2, indicates a clear area for potential improvement in the model’s assessment capabilities.

Criterion 5, evaluating “The LLM’s effective case summarization and clear presentation to the doctor”, achieved the second-highest score at 4.44. This score did not significantly differ from that of Criterion 1 (ANOVA and Tukey post hoc test, *p* = 0.634), suggesting that the surgeons highly regarded the AI’s case presentations. 

Criterion 6, “The LLM did the patient history just as well as I would have done it”, achieved a score of 4.39, statistically comparable to Criterion 1 (ANOVA and Tukey post hoc test, *p* = 0.4676). This suggests that the AI’s skills in taking patient history are on par with those of human practitioners.

**Figure 3:**
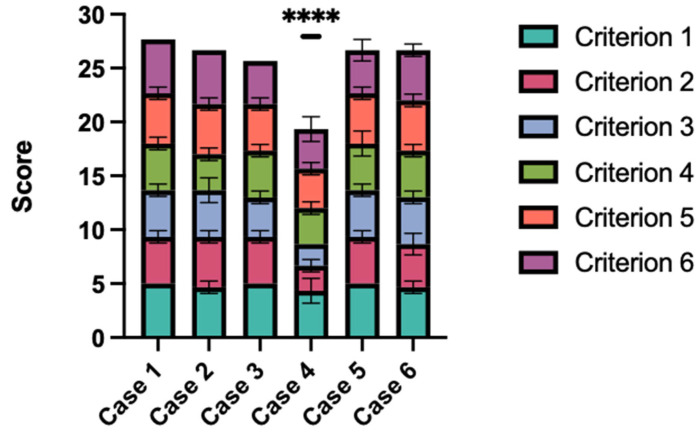
Scores.

The language model achieved an overall average score of 4.24 across all scenarios, corresponding to a cumulative average score of 25.44 (Figure 3). Case 4 received the lowest evaluation, with an average rating of 3.22 and a total score of 19.33. While Case 4’s scores were statistically lower than those of all other cases (*p* < 0.0001), there were no significant statistical differences in the scores among the remaining cases. 

GPT-4’s responses were evaluated using a Likert scale based on the six criteria seen in Table 1. This graph shows the average score for each case, where 30 is the highest possible score and 6 is the lowest. Case 4 was rated statistically significantly worse (*p* < 0.0001).

## 4. Discussion

ChatGPT, since its introduction, has seen an unprecedented rate of adoption, achieving a milestone of one million users within just five days post-launch. Currently, its user base exceeds 180 million, with openai.com garnering around 1.5 billion monthly visits, establishing it as the most rapidly expanding application to date [29]. The evolution of OpenAI’s technologies has been swift, with the GPT-4 model not only processing text but also possessing capabilities for image analysis, user interaction through speech, and image generation [30,31].

The remarkable functionalities of LLMs have spurred an increase in research within the medical sector, exploring their potential applications. Despite the impressive performances showcased in these studies, there is a consensus among many researchers that AI is not yet fully equipped for clinical deployment [32,33,34]. A significant aspect, often underutilized or minimally employed in these studies, is prompt engineering—an element crucial for enhancing LLM performance. Addressing this underutilization, Meskó recently published a prompt engineering tutorial tailored for medical professionals, highlighting its importance [14].

Also, the effective interaction with Lipo-GPT was contingent on the application of prompt engineering. This study highlights how the true capabilities of LLMs can be swiftly underestimated without such crucial techniques.

Examining the ratings reveals that Case 4 is notably poorly evaluated (Figure 3). In this instance, Lipo-GPT incorrectly identified a clear case of obesity as lipedema, as per the human evaluators’ assessment. This outcome is intriguing, especially considering that physicians without experience in treating lipedema often fail to recognize it, typically mistaking it for obesity—a direct contrast to the AI’s error in this case [20]. Interestingly, Lipo-GPT demonstrated markedly fewer difficulties in identifying lymphedema and vascular insufficiencies, as evidenced in Cases 5 and 6. The reasons behind this observation are speculative, as the specific sources of information utilized by the AI in processing these cases are unknown. It is conceivable that the AI’s training data might contain limited texts delineating the distinctions between lipedema and obesity. Given that obesity is a crucial differential diagnosis for lipedema and even seasoned doctors can struggle to distinguish between the two, this could be a contributing factor. Additionally, it is possible that the prompt engineering may have overly biased Lipo-GPT towards diagnosing “lipedema”, suggesting that a variation in prompting could potentially enhance its differential diagnostic capabilities [35].

Upon examining the evaluated criteria, it becomes apparent that traditional medical skills, such as diagnosing, planning further diagnostic procedures, and determining surgical indications, received lower ratings. This outcome aligns with expectations, considering that such skills in humans are honed through years of intensive study and clinical experience. Exploring the potential of AI’s image recognition function to enhance diagnostic accuracy presents an intriguing prospect.

Conversely, the AI demonstrated reliable proficiency in obtaining patient medical histories and presenting cases to medical staff in a clear, concise format. This efficiency was so notable that the evaluating doctors concurred that the AI’s medical history taking was on par with their own. Given the critical importance of time management in today’s high-pressure clinical environment, where increasing time is devoted to documentation and both doctors and patients often feel the constraint of limited interaction time, the assistance of an AI tool in medical history gathering and documentation could be a significant cost-efficient asset.

It is important to note that the development of GPT-4 was geared towards general cognitive abilities rather than being tailored specifically for healthcare applications. The training of this model relied exclusively on data accessible from public internet sources [3]. Given that GPT-4 was not explicitly trained for plastic surgery or the treatment of lipedema, it is reasonable to predict that similar levels of success could be attained in other medical specialties through the application of prompt engineering. A notable limitation of GPT-4 is that its database does not update in real time and, at the moment, only contains information up to September 2021, indicating room for improvement in its performance with more recent and higher-quality data. 

A limitation of this study is its reliance on only six simulated scenarios. This limited scope may not adequately represent the full spectrum of lipedema cases, potentially affecting the generalizability of our findings to all clinical conditions. This study compared GPT-4’s performance against the evaluating doctors’ expectations, but it did not involve a direct comparison between the AI’s performance and that of human physicians under similar scenarios. Future studies could enhance the reliability of findings by including such comparative analyses, thereby providing a clearer perspective on the AI’s practical efficacy relative to human clinicians in clinical settings. Additionally, this study was limited to using six criteria for evaluating GPT-4’s performance. Expanding these criteria to encompass more specific aspects of lipedema diagnosis and treatment could yield a more comprehensive and detailed assessment of the model’s capabilities. Another limitation is that this study was conducted using simulated scenarios, albeit based on realistic examples, rather than real patient interactions, which may affect the applicability of the findings to actual clinical practice.

While this study effectively demonstrated the potential advantages of employing AI in the management of lipedema, it is important to recognize the potential risks and limitations associated with AI reliance in clinical decision making. Careful consideration must be given to the fact that AI systems, such as GPT-4, may not fully replicate the complex clinical judgment exercised by human physicians. Additionally, AI’s decisions, influenced by training data, may carry inherent biases if the data are unrepresentative or outdated. 

Given that this study utilized simulated scenarios, it did not explore the ethical considerations, such as privacy, informed consent, and potential biases inherent in AI algorithms. Future research should rigorously address these ethical aspects to ensure the responsible deployment of AI technologies in actual healthcare settings [36].

Further research is necessary to assess the sustainability and economic viability of integrating AI technologies like GPT-4 in routine clinical practice. Such studies would provide valuable insights into how AI can enhance or alter long-term treatment efficacy and cost-effectiveness in managing complex conditions like lipedema.

In summary, our assessment of the current technological capabilities suggests that GPT-4 can already reliably handle routine tasks if it is guided by prompting. It remains to be seen whether future enhancements will enable the support of more complex medical activities. Given the rapid advancement of AI systems, such progression appears to be a matter of when, not if. It has become a crucial task for physicians to understand the functioning of AI systems, along with their potential opportunities and risks. The objective should be to utilize AI for efficiently managing routine tasks, thereby freeing up more time for physicians to focus on direct patient care. While AI will probably never supplant a trained doctor, this study illustrates a practical application of how AI could enhance the overall treatment process.

## Figures and Tables

**Table 1:** Likert scale.

Grading Scale						
	Criteria	1 Strongly Disagree	2 Disagree	3 Neither Agree or Disagree	4 Agree	5 Strongly Agree
**Criterion 1**	The large language model (LLM) correctly understood and captured the issue.					
**Criterion 2**	The LLM correctly stated the most likely diagnosis.					
**Criterion 3**	The LLM mentioned the correct recommendations for further diagnostic steps.					
**Criterion 4**	The LLM correctly assessed whether surgery is indicated.					
**Criterion 5**	The LLM summarized the case well and presented it satisfactorily and clearly to the doctor.					
**Criterion 6**	The LLM did the patient history just as well as I would have done it.					

## Data Availability

The complete data set can be downloaded from the Supplementary Materials.

## Supplementary Materials

The following supporting information can be downloaded at: https://www.mdpi.com/article/10.3390/life14050646/s1. Bewertung Mean.xlsx.
